# Exome Sequencing in *BRCA1-* and *BRCA2*-Negative Greek Families Identifies *MDM1* and *NBEAL1* as Candidate Risk Genes for Hereditary Breast Cancer

**DOI:** 10.3389/fgene.2019.01005

**Published:** 2019-10-18

**Authors:** Stavros Glentis, Alexandros C. Dimopoulos, Konstantinos Rouskas, George Ntritsos, Evangelos Evangelou, Steven A. Narod, Anne-Marie Mes-Masson, William D. Foulkes, Barbara Rivera, Patricia N. Tonin, Jiannis Ragoussis, Antigone S. Dimas

**Affiliations:** ^1^Division of Molecular Biology and Genetics, Biomedical Sciences Research Center Al. Fleming, Vari, Greece; ^2^Department of Hygiene and Epidemiology, University of Ioannina Medical School, Ioannina, Greece; ^3^Department of Epidemiology and Biostatistics, Imperial College London, London, United Kingdom; ^4^Dalla Lana School of Public Health, University of Toronto, Toronto, ON, Canada; ^5^Women's College Research Institute, Women’s College Hospital, Toronto, ON, Canada; ^6^Centre de recherche du Centre hospitalier de l’Université de Montréal and Institut du cancer de Montréal, Montreal, QC, Canada; ^7^Department of Oncology, McGill University, Montreal, QC, Canada; ^8^Lady Davis Institute for Medical Research, Jewish General Hospital, Montreal, QC, Canada; ^9^Department of Medical Genetics, The Research Institute of the McGill University Health Centre, Montreal, QC, Canada; ^10^Department of Medicine, McGill University, Montreal, QC, Canada; ^11^Department of Human Genetics, McGill University, Montreal, QC, Canada; ^12^Cancer Research Program, The Research Institute of the McGill University Health Centre, Montreal, QC, Canada; ^13^McGill University and Genome Quebec Innovation Centre, Montreal, QC, Canada

**Keywords:** hereditary breast cancer, exome sequencing, Greek population, candidate risk variants, *MDM1*, *NBEAL1*

## Abstract

Approximately 10% of breast cancer (BC) cases are hereditary BC (HBC), with HBC most commonly encountered in the context of hereditary breast and ovarian cancer (HBOC) syndrome. Although thousands of loss-of-function (LoF) alleles in over 20 genes have been associated with HBC susceptibility, the genetic etiology of approximately 50% of cases remains unexplained, even when polygenic risk models are considered. We focused on one of the least-studied European populations and applied whole-exome sequencing (WES) to 52 individuals from 17 Greek HBOC families, in which at least one patient was negative for known HBC risk variants. Initial screening revealed pathogenic variants in known cancer genes, including *BARD1*:p.Trp91* detected in a cancer-free individual, and *MEN1*:p.Glu260Lys detected in a BC patient. Gene- and variant-based approaches were applied to exome data to identify candidate risk variants outside of known risk genes. Findings were verified in a collection of Canadian HBOC patients of European ancestry (FBRCAX), in an independent group of Canadian BC patients (CHUM-BC) and controls (CARTaGENE), as well as in individuals from The Cancer Genome Atlas (TCGA) and the UK Biobank (UKB). Rare LoF variants were uncovered in *MDM1* and *NBEAL1* in Greek and Canadian HBOC patients. We also report prioritized missense variants *SETBP1*:c.4129G > C and *C7orf34*:c.248C > T. These variants comprise promising candidates whose role in cancer pathogenicity needs to be explored further.

## Introduction

Breast cancer (BC) is the most common type of cancer in women worldwide, with almost 1.7 million new cases and 500,000 deaths annually (Ferlay et al., 2015). In Europe alone, there are 464,000 new cases and 131,000 deaths annually, but the age-standardized incidence rates across countries vary threefold (Ferlay et al., 2013). Greece, with 6,000 cases annually, displays one of the lowest BC rates among European populations. This, however, is likely to be an underestimate arising from under-reporting of cases (Ferlay et al., 2013). Although variation in incidence rates of BC is driven by both genetic and environmental factors (Garcia-Closas et al., 2014), approximately 10% of BC cases have a strong genetic basis (Lynch et al., 2008; King-Spohn and Pilarski, 2014; Terui-Kohbata and Yoshida, 2017). These cases, which show strong familial clustering, or are characterized by early onset of disease, are termed hereditary and are most commonly encountered in the context of hereditary breast and ovarian cancer (HBOC) syndrome (Lynch et al., 2008; Terui-Kohbata and Yoshida, 2017). This syndrome is primarily characterized by increased risk of BC and of ovarian cancer (OC), but also of other cancers, including pancreatic and prostate cancers (Solomon et al., 2012). BC can also manifest in other inherited cancer syndromes, including Cowden, Li-Fraumeni, Peutz-Jeghers, and hereditary gastric cancer syndromes (Rahman, 2014).

Disease-associated genetic variants located in two major predisposition genes, *BRCA1* and *BRCA2*, account for approximately 25% of HBOC cases worldwide (Kast et al., 2016). Heterozygote individuals for pathogenic variants in BRCA1 have a 72% and 44% increased risk for BC and OC, respectively, by age 80 years. In the case of BRCA2, equivalent risks for BC and OC by age 80 years amount to 69% and 17% (Kuchenbaecker et al., 2017). Individuals harboring pathogenic variants in these genes also have increased risk for other malignancies, including melanoma, prostate, and pancreatic cancer, which suggests a broader role for these genes in cancer predisposition (Levy-Lahad and Friedman, 2007). Thousands of different loss-of-function (LoF) pathogenic variants have been identified in *BRCA1* and *BRCA2* across different populations, the majority of which are individually rare (Mcclellan and King, 2010). In the Greek population, *BRCA1* and *BRCA2* pathogenic variants account for 27.9% of HBOC cases nationally, with over half (58.5%) of these arising from six principal founder variants (Konstantopoulou et al., 2014).

In addition to pathogenic variants in *BRCA1* and *BRCA2*, moderate- to high-penetrance LoF variants in at least 24 genes have been linked to BC susceptibility (Nielsen et al., 2016). However, of these, only *ATM*, *CDH1*, *CHEK2*, *NF1*, *PALB2*, *PTEN*, *STK11*, and *TP53* are established risk genes, with the remaining candidates lacking reliable effect estimates to date (Easton et al., 2015). The majority of these genes have roles in biological pathways linked to genome maintenance, although other functions including cell adhesion (*CDH1*), RAS signaling (*NF1*), and PI3K/AKT/mTOR signaling (*PTEN*) are reported (Nielsen et al., 2016). Similar to pathogenic variants in *BRCA1* and *BRCA2*, LoF alleles in these genes may also predispose to other forms of cancer (Solomon et al., 2012). In addition to BC arising from the effects of single highly penetrant variants, susceptibility to disease can also be explained by the presence of multiple, low-penetrance alleles. Population-level genome-wide association studies (GWAS) have highlighted at least 150 such BC susceptibility loci to date, which explain ∼18% of the familial relative risk (Michailidou et al., 2017).

Despite extensive research, approximately 50% of HBOC cases remain of unknown genetic etiology, even when polygenic risk scores are taken into consideration (Michailidou et al., 2017; Mavaddat et al., 2019). Applying whole-exome sequencing (WES) to HBOC families with unknown genetic etiology is an approach that has contributed to the identification of novel disease-predisposing variants (Thompson et al., 2012; Kiiski et al., 2014; Cybulski et al., 2015; Rivera et al., 2017). This strategy is expected to yield additional pathogenic variants, especially when applied to lesser-studied populations, such as that of Greece. Risk alleles segregating at extremely low frequencies in other populations may exist in slightly higher frequencies in Greek patients due to population-specific effects. In the present study, we applied WES to Greek HBOC families of unknown genetic etiology to identify novel BC susceptibility variants. Findings were followed up in a collection of Canadian HBOC patients of European ancestry (FBRCAX) (Cybulski et al., 2015; Rivera et al., 2017) and subsequently validated in an independent BC patient group from the Centre Hospitalier de l’Université de Montréal (CHUM-BC) and control population (CARTaGENE) (Awadalla et al., 2013). Findings were also explored in cancer patients from The Cancer Genome Atlas (TCGA) (www.cancergenome.nih.gov and the UK Biobank (UKB) (Sudlow et al., 2015). All data sets interrogated in the present study are summarized in **Supplementary Table 1**.

## Materials and Methods

### Patient Screening and Selection of Study Subjects

#### Greek Breast Cancer (GRBC) Study

BC and OC patients were screened for known cancer risk variants at the molecular diagnostics laboratory at National Centre of Scientific Research (NCSR) Demokritos. Index patients were those individuals who developed BC or OC before the age of 40 years or had at least two first-degree relatives who were also diagnosed with cancer. Screening involved Sanger sequencing (exons and intron/exon boundaries) and multiplex ligation-dependent probe amplification (MLPA) analysis of *BRCA1* and *BRCA2*. Patients who were negative for known disease-associated variants in *BRCA1* and *BRCA2*, were further screened for single-nucleotide variants (SNVs) and indels in 94 cancer-predisposing genes on the Illumina TruSight Cancer Panel (**Supplementary Note 1**). Patients, who were negative for known causal variants and their informative relatives (including family members affected by any form of cancer, parents of index patients, and obligate carriers), were subjected to WES and comprised the Greek BC study (GRBC, 47 females and 5 males from 17 HBOC families). Of the selected individuals, 30 were BC and 3 were OC patients (**Supplementary Table 2**). Written informed consent was obtained from all individuals prior to genetic testing, and the study was approved by the bioethics committee of NCSR Demokritos (240/EH/11.3, updated Feb. 14, 2014) in agreement with the 1975 Helsinki statement.

#### FBRCAX Study

We subsequently explored initial findings in 51 HBOC index patients of FBRCAX from Cybulski et al. (2015) and Rivera et al. (2017), who were negative for disease variants in *BRCA1* and *BRCA2, CHEK2, NBN*, and *PALB2*. Exome capture, sequencing, and raw data analysis methods have been described previously (Cybulski et al., 2015) and are summarized in **Supplementary Note 2**. WES data from FBRCAX were screened to confirm absence of other known disease-associated variants (94 cancer genes; Illumina TruSight Cancer Panel).

#### CHUM-BC Study

Our study also included an independent group of female BC patients from Centre Hospitalier de l’Université de Montréal (CHUM-BC) (N = 512) who were diagnosed with invasive BC at <65 years of age. CHUM-BC patients were defined as French Canadian based on review of last names.

#### CARTaGENE Study

We also included a collection of cancer-free individuals of French-Canadian origin (N = 1,940: 970 women, 970 men) recruited through CARTaGENE. CARTaGENE individuals were born in Quebec, spoke French as their first language, and their parents and all four grandparents were born in Canada. They ranged in age from 45 to 65 years (average, 53 years), had no personal history of cancer, and no documented cancer cases in first-degree relatives.

### Exome Sequencing and Variant Calling

Exome capture on gDNA was performed using the Ion Targetseq™ exome enrichment kit, and samples were sequenced on an Ion Proton (IP) platform (Thermo Fisher Scientific Inc., West Palm Beach, FL, USA) at the Genomics Facility of the Biomedical Sciences Research Center (BSRC) Alexander Fleming. Variants (SNVs and indels) were called from IP raw data using two software packages: the IP built-in Torrent Variant Caller (TVC v5.0) and the Genome Analyzer Toolkit (GATK) (Depristo et al., 2011). Copy number variants (CNVs) were called using eXome-Hidden Markov Model (XHMM) software, with default options (Fromer et al., 2012). Additional WES on an Illumina platform was performed for seven GRBC individuals to compare IP-generated data to data derived from the widely used Illumina platform, which employs different chemistry (Boland et al., 2013) (**Supplementary Note 2**).

### Kinship Analysis and Population Structure

Pairwise kinship analysis for all GRBC individuals was performed using vcftools (Danecek et al., 2011) to confirm reported pedigrees, to flag potential errors, and to reveal hidden genetic relationships. Principal component analysis (PCA) was carried out using EIGENSTRAT (Price et al., 2006) to determine the extent of population structure and to compare GRBC individuals with individuals from the 1,000 genome populations (Genomes Project et al., 2015).

### Gene-Based Prioritization and Validation in Independent Patient Groups

**Figure 1:**
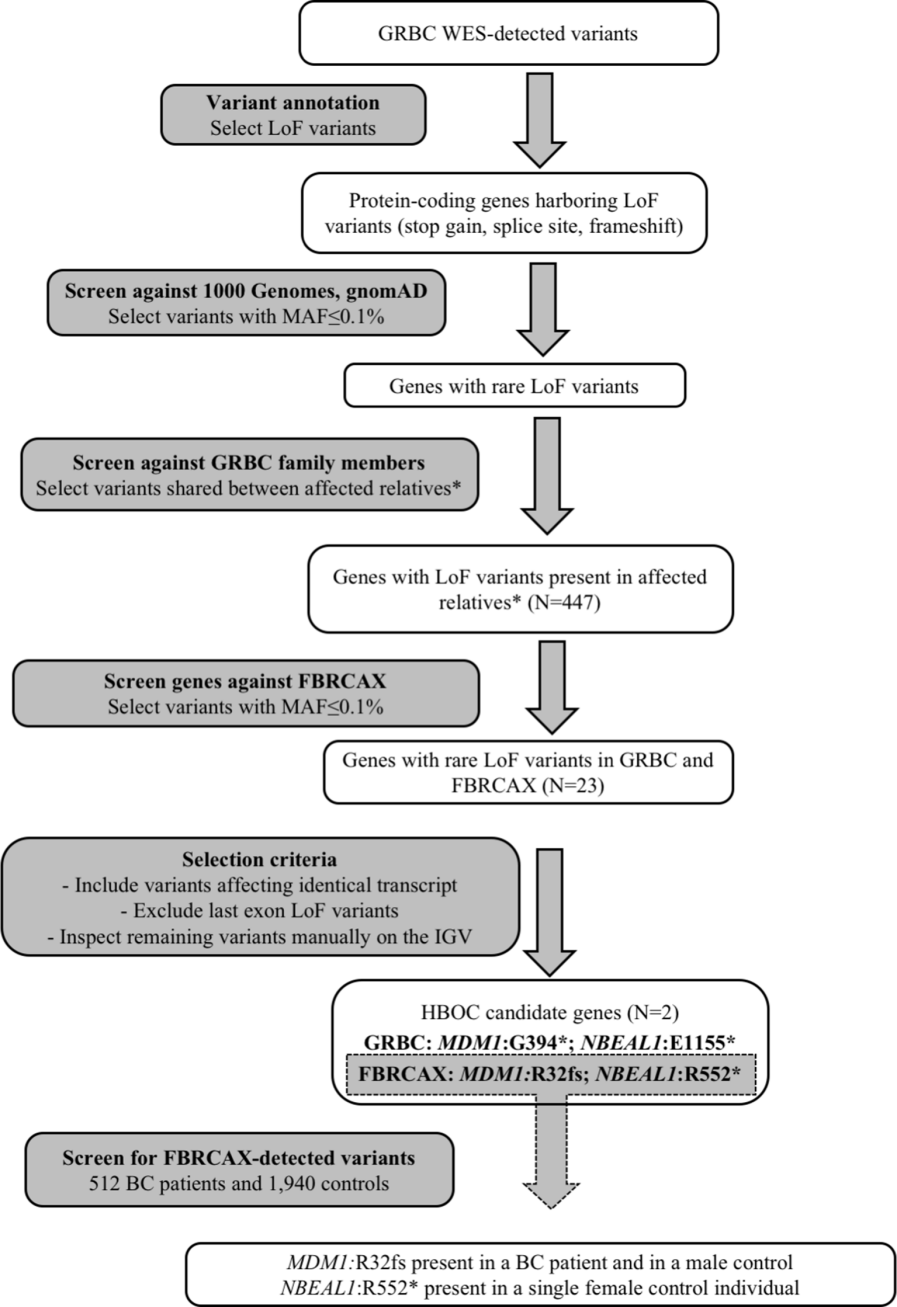
Gene-based prioritization workflow. *For families where this was possible to be ascertained.

To identify candidate HBOC susceptibility loci, we applied a gene-based shortlisting approach to the total of called variants in GRBC. Our approach focused on genes that harbored LoF variants (stop-gain, essential splice site, and frameshift), because these variant types are the most likely to have functional impact and are commonly linked to disease susceptibility (Richards et al., 2015). We limited our search to rare variants (minor allele frequency [MAF] ≤ 0.1% in both 1,000 genomes (Genomes Project et al., 2015) and gnomAD (Lek et al., 2016) global populations) that were present in at least two GRBC patients from a single family, where this was possible to be ascertained (**Figure 1** and **Supplementary Note 3**). Shortlisted variants were inspected visually on the Integrative Genomics Viewer (IGV) to exclude possible false positives (Robinson et al., 2017). A subset of shortlisted variants was re-sequenced and experimentally validated using AmpliSeq (Thermo Fisher Scientific Inc., West Palm Beach, FL, USA) (**Supplementary Note 4**).

Genes harboring shortlisted variants were considered candidate risk genes and were followed up in the FBRCAX collection of patients (N = 51). In FBRCAX, we examined the distribution of rare LoF variants in these prioritized genes. We focused on genes that harbored the same or a different rare LoF variant in at least one FBRCAX patient, restricting our analysis to variants mapping on the same transcript. Genes harboring at least one rare LoF variant in both GRBC and FBRCAX and meeting criteria pertaining to MAF, transcript, and position in gene were further shortlisted as likely risk candidates (**Supplementary Note 3**). Variants in FBRCAX were validated experimentally through Sanger sequencing (**Supplementary Note 4**). We further examined the presence of FBRCAX-detected candidate variants in female BC patients from CHUM-BC and in cancer-free individuals from CARTaGENE (**Supplementary Note 5**).

To evaluate the possible involvement of shortlisted genes in general cancer susceptibility, we explored large publicly available data sets and performed burden analysis. LoF variant cumulative frequencies were compared across non–Finnish European cancer patients from TCGA (NFE-TCGA), and NFE individuals from the Exome Aggregation Consortium (ExAC) (Lek et al., 2016) after excluding TCGA patients (NFE-ExAC-nonTCGA) (**Supplementary Note 6**). We applied the sequence kernel association test (SKAT) (*p* < 0.05) (Wu et al., 2011) with inclusion of variants with MAF ≤ 0.1% in both groups. Last-exon variants as well variants with MAF ≥ 0.1% in either 1000 Genomes or gnomAD were excluded.

### Variant-Based Prioritization and Verification in Independent Samples

To complement the gene-based variant prioritization strategy, we also applied three variant-based prioritization approaches to identify candidate BC risk variants in GRBC. Through these approaches, in addition to LoF variants, we also examined in-frame indels, missense, and stop-loss variants (**Supplementary Note 7** and **Supplementary Figure 1**). The three approaches addressed: a) variants that mapped in any of 1,580 genes with a role in cancer (cancer gene variants [CGV]; **Supplementary Note 8**, and **Supplementary Table 3**), b) identical variants shared across unrelated patients, or different variants mapping to the same gene in unrelated patients (shared variants/genes in unrelated [SVGU]), and c) ultra-rare variants (MAF < 0.01%) present in a single patient or family (family-specific variants [FSV]). We subsequently considered variants prioritized by one, two or all three of the above approaches, and that fulfilled criteria for MAF, predicted pathogenicity as predicted by *in silico* tools, and presence in affected relatives (**Supplementary Note 7**). Following visual inspection on the IGV and experimental validation of a subset of variants (**Supplementary Note 4**), we explored the presence of shortlisted variants in FBRCAX.

To further evaluate the variants prioritized through the above approach, we explored their presence across independent groups of cancer patients and cancer-free individuals. We examined the following groups: a) NFE-TCGA cancer patients, b) NFE-ExAC-nonTCGA cancer-free individuals, c) UKB cancer patients of European ancestry (EUR-UKB cancer patients), and d) UKB cancer-free individuals of European ancestry (EUR-UKB cancer free individuals). In the case of EUR-UKB cancer patients, we considered: i) all cancer patients, and ii) female BC patients (**Supplementary Note 6**).

To investigate the possible role of variants shortlisted through the above approach in general cancer susceptibility, we used Fisher’s exact test (FET) (two-sided *p* < 0.05) to compare allele frequencies across: a) NFE-TCGA (cancer patients) versus NFE-ExAC-nonTCGA (cancer-free individuals), b) EUR-UKB cancer patients versus EUR-UKB cancer-free individuals, and c) EUR-UKB female BC patients versus EUR-UKB female cancer-free individuals (**Supplementary Note 9**).

## Results

**Figure 2:**
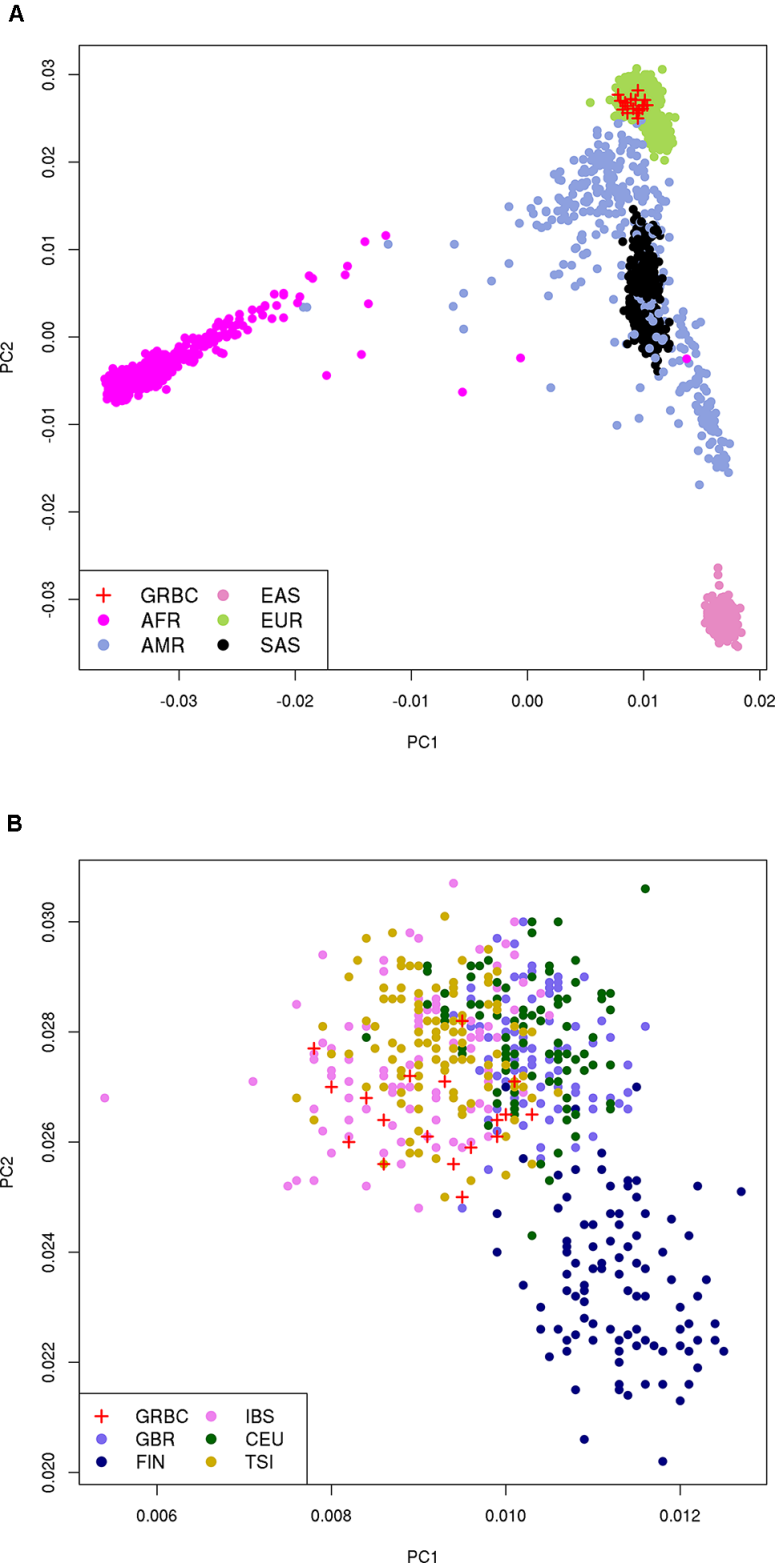
Principal component analysis (PCA) on 27,666 coding variant positions reveals that GRBC patients (one patient per family) map close to: **(A)** the EUR superpopulation, and **(B)** the IBS and TSI populations of 1000 Genomes. Abbreviations: GRBC, Greek Breast Cancer study; AFR, African; AMR, Admixed American; EAS, East Asian; EUR, European; SAS, South Asian; CEU, Central European; TSI, Tuscan; FIN, Finnish; GBR, British; IBS, Iberian.

We report a mean coverage depth of 154× for targeted sequenced regions (range, 71–210×, **Supplementary Tables 4 and 5**). On average, 33,048 SNVs, 1,212 indels, and 30 CNVs were detected per GRBC individual (**Supplementary Table 6**). For IP-sequenced samples, we report an average overlap of 90.1% for SNVs called using TVC versus GATK (**Supplementary Table 7**). Comparison across sequencing platforms (IP vs. Illumina) revealed average overlaps of 85.7% (SNVs) and 48.8% (indels) (**Supplementary Note 10** and **Supplementary Tables 8 and 9**). Kinship analysis confirmed patient-reported relationships in 16 out of 17 pedigrees. A single BC patient (F14S01) was found to be genetically unrelated to her reported mother and sister (relatedness ϕ = −0.019 and −0.015, respectively) and to all other GRBC individuals and was excluded from subsequent analyses. PCA revealed that GRBC unrelated patients map close to populations of European ancestry, particularly to the Iberian (IBS) and Tuscan (TSI) 1000 Genomes populations (Genomes Project et al., 2015) (**Figure 2**). Although this finding derives from coding variants only, it is in line with work reporting genetic similarity between Greek and Italian subpopulations (Stamatoyannopoulos et al., 2017).

### Variants in Known BC Risk Genes

In our effort to identify novel HBOC risk loci, we first sought to ascertain whether known risk variants were present in GRBC non-index patients, or in unaffected individuals. A private stop-gain variant (not reported in public databases to date) in exon three of *BARD1* (ENST00000260947:c.273G > A, p.Trp91*) was detected in a single unaffected female individual (F14S03; age, > 60 years). Of note, the variant was not present in the carrier’s daughter, who developed BC at 37 years of age. *BARD1* encodes a ligase that forms a heterodimer with *BRCA1* and is involved in DNA repair (Shakya et al., 2008). Accumulating evidence suggests that LoF variants in *BARD1* may elevate the risk for BC (Castera et al., 2018). We also detected an established pathogenic variant for multiple endocrine neoplasia type 1 (MEN-1) syndrome, in *MEN1* (ENST00000337652:c.778G > A, p.Glu260Lys, rs104894268) (Teh et al., 1998) in a non-index patient, diagnosed with BC and primary parathyroid hyperplasia at 50 years. Given that MEN-1 syndrome predisposes to BC and to parathyroid adenomas (Dreijerink et al., 2014), this variant may have contributed to the development of both malignancies. However, it was not detected in the carrier’s niece who developed BC at 39 years of age, suggesting that disease risk in these two related patients may have different underlying genetic etiologies.

### Experimental Validation of Shortlisted Variants

Gene- and variant-based prioritization approaches yielded a total of 468 (mapping in 447 genes) and 1,844 variants, respectively. To experimentally validate shortlisted variants, we re-sequenced a subset of 148 SNVs and confirmed the presence of 140 (validation rate 94.6%, **Supplementary Note 4** and **Supplementary Table 10**).

### Genes Prioritized for BC Susceptibility

Gene-based prioritized LoF variants were present in GRBC index patients and in at least one affected relative. Of the 447 genes harboring these variants, two (*MDM1* and *NBEAL1*) also harbored at least one rare LoF variant in FBRCAX (**Figure 1** and **Table 1**). LoF variants in *MDM1* and *NBEAL1* mapped on the same transcript in GRBC and FBRCAX, did not reside in the last exon, and their presence was confirmed experimentally through AmpliSeq (GRBC) and Sanger sequencing (FBRCAX). For GRBC cases where family pedigrees were available, we confirmed that the reported LoF variant (for both *MDM1* and *NBEAL1*) was present in all related patients examined. Burden analysis did not reveal LoF variant enrichment for either gene in NFE-TCGA cancer patients (**Supplementary Table 11**), but this likely arises in part from the fact that TCGA includes patients with different cancer types.

**Table 1:** Genes with rare LoF variants in individuals from GRBC, FBRCAX, CHUM-BC, and CARTaGENE.

Gene	Nucleotide change	Protein change	rsid (v147)	GRBC carriers	GRBC families	FBRCAX carriers	CHUM-BC carriers	CARTaGENE carriers	MAF gnomAD
*MDM1*	c.1180G > T	p.G394X	.	2	1	-	n/a	n/a	.
	c.98dupA	p.R32fs	rs1339213002	-	-	1	1	1	0.0000203
*NBEAL1*	c.3463G > T	p.E1155*	rs200689887	2	1	-	n/a	n/a	0.0006
	c.1654C > T	p.R552*	rs921590150	-	-	1	-	1	0.0000128

GRBC, Greek Breast Cancer study; FBRCAX, French-Canadian HBOC patients; CHUM-BC, breast cancer patients from Centre Hospitalier de l’Université de Montréal; CARTaGENE, CARTaGENE research platform; LoF, loss-of-function; “-”,variant was not detected; n/a, not applicable because variant not further interrogated in CHUM-BC and CARTaGENE as it was not detected in FBRCAX.

**Figure 3:**
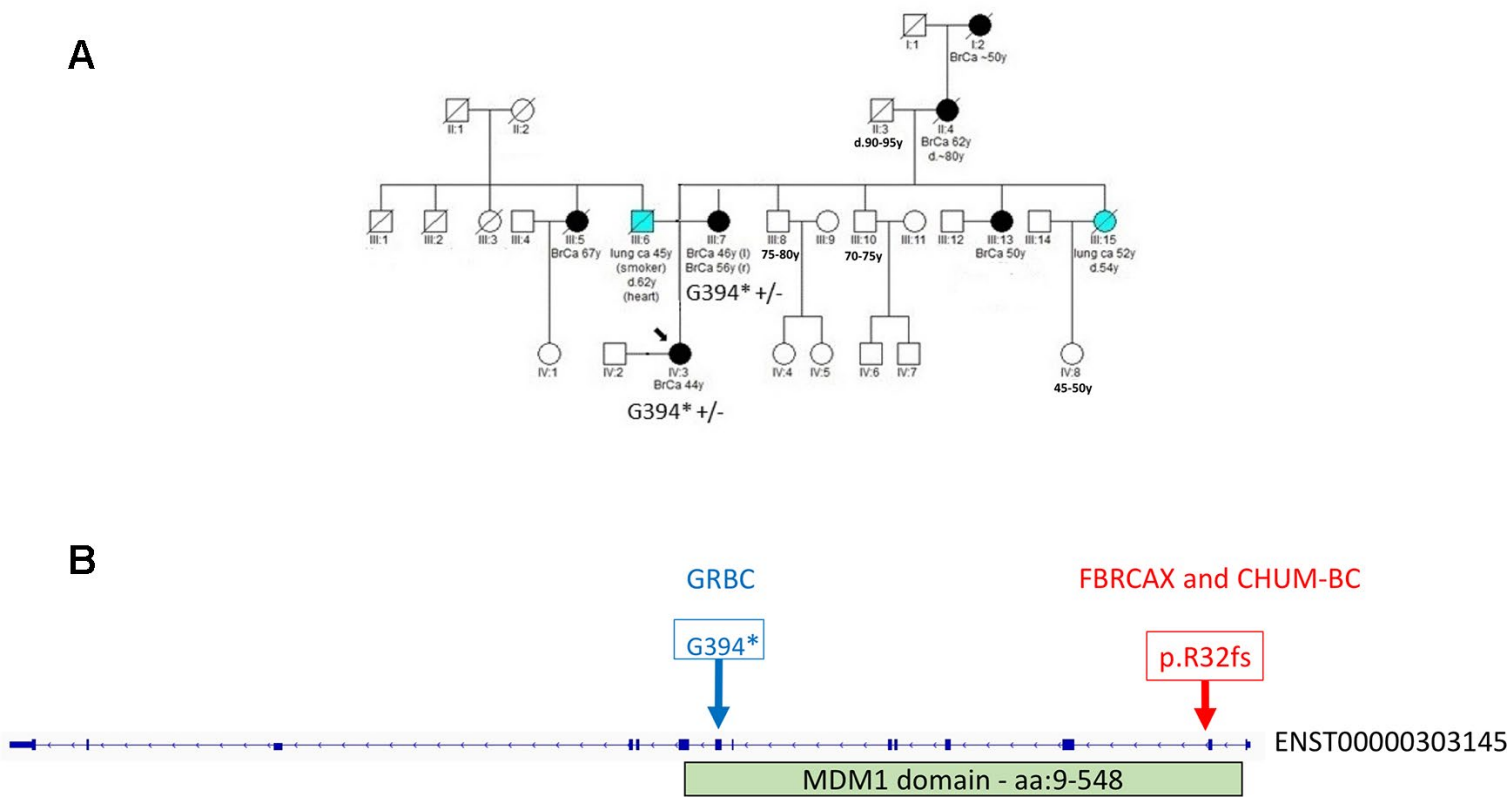
**(A)** Segregation of *MDM1*:G394* in GRBC pedigree F25. Both mother (diagnosed with bilateral BC at 46 and 56 years of age) and daughter (diagnosed with BC at 44 years of age) were heterozygous carriers of the *MDM1* stop-gain variant. **(B)** Genomic positions of *MDM1* LoF variants detected in Greek (GRBC, *MDM1*:G394*) and French Canadian (FBRCAX and CHUM-BC, *MDM1*:p.R32fs) patients. Variants were experimentally validated using Ampliseq (GRBC) and Sanger sequencing (FBRCAX). CHUM-BC variants were detected using the iPLEX MassARRAY.

In the case of *MDM1*, variants *MDM1*:p.G394* and *MDM1*:p.R32fs were initially detected in GRBC and FBRCAX, respectively. Notably, *MDM1*:p.R32fs was also detected in a CHUM-BC patient. These variants localize on opposite ends of the same functional domain (**Figure 3**). Stop-gain variant *MDM1*:p.G394* maps on a splice site and may thus exert additional functional effects. *MDM1*:p.R32fs (gnomAD MAF = 0.002%) is adjacent to a position where two other distinct LoF variants, that affect the same amino acid, have been reported (gnomAD cumulative MAF = 0.0016%), but whose phenotypic impact remains to be described. *MDM1*:p.G394* was detected in a GRBC mother-daughter pair, diagnosed, respectively, with bilateral BC (at 46 and 56 years) and BC at 44 years. This variant was absent in all other GRBC individuals and has not been reported in public databases to date. *MDM1*:p.R32fs was present in two French-Canadian BC patients, one each from FBRCAX (out of 51 patients) and CHUM-BC (out of 512 patients). The *MDM1* carrier in FBRCAX developed BC at the age of 47 years and had a strong family history of cancer, including five cases of BC and one case each of esophageal and bladder cancer. The *MDM1* carrier from CHUM-BC, who was negative for *BRCA1*, *BRCA2*, and *PALB2* pathogenic variants, developed BC at the age of 51 years. For this individual there is no information on cancer family history. *MDM1*:p.R32fs, was also detected in a single French-Canadian male individual from CARTaGENE (of 1,924 individuals) (**Supplementary Note 4**) who was cancer-free at the age of 65 years. *MDM1* is an evolutionary conserved gene encoding a protein that binds and stabilizes microtubules, and acts as a negative regulator of centriole duplication (Van De Mark et al., 2015). It is expressed in multiple tissues, including breast, and is predicted to be intolerant to homozygous/double heterozygous LoF variants (ExAC pRec score = 0.96, LoF_Z score = 2.15) (Lek et al., 2016). At the somatic mutation level, *MDM1* point mutations and copy number alterations (CNA) are present in 3% of 817 BC tumors of the TCGA data set (**Supplementary Figure 2**). These mutations tend to co-occur with *BRCA2* mutations (*p* = 0.002) (**Supplementary Table 12**).

**Figure 4:**
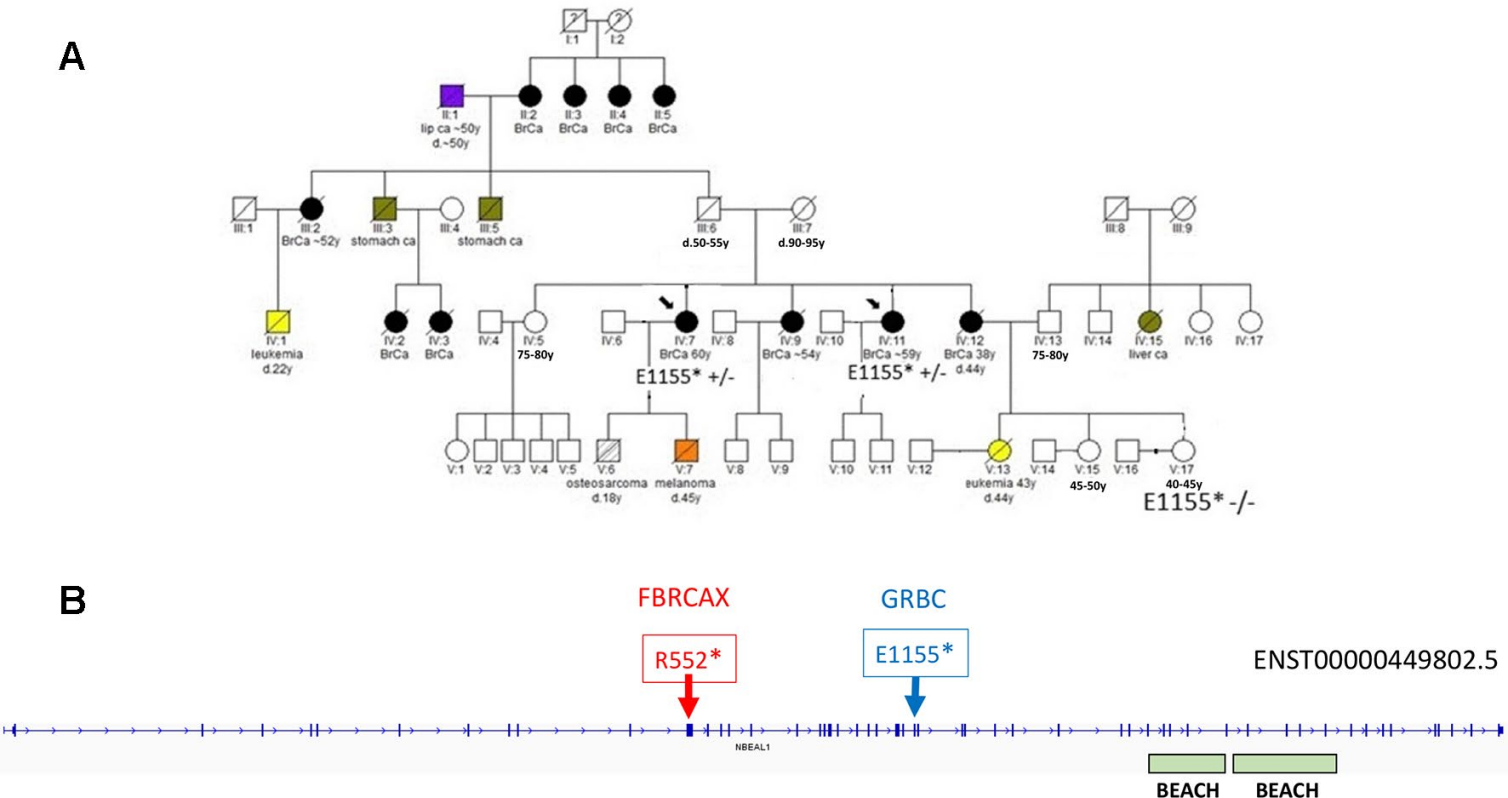
**(A)** Segregation of *NBEAL1*:E1155* in GRBC pedigree F22. Two sisters (diagnosed with BC at 59 and 60 years) were heterozygous carriers of the *NBEAL1* stop-gain variant. Their niece, who was cancer-free at 40 years of age, was not a carrier of the variant. **(B)** Genomic positions of *NBEAL1* LoF variants detected in GRBC (*NBEAL1*:E1155*) and FBRCAX (*NBEAL1*:p.R552*) patients. Variants were experimentally validated using Ampliseq (GRBC) and Sanger sequencing (FBRCAX).

In the case of *NBEAL1*, variants *NBEAL1*:p.E1155* and *NBEAL1*:p.R552* were detected in GRBC and FBRCAX, respectively (**Figure 4**). *NBEAL1*:p.E1155* (gnomAD MAF = 0.06%) was present in two GRBC affected sisters, diagnosed with BC at 59 and 60 years. In FBRCAX, *NBEAL1*:p.R552* (gnomAD MAF = 0.001%) was present in a single patient (of 51 patients), who developed BC at the age of 23 years. *NBEAL1*:p.R552* was also detected in a single female individual from CARTaGENE (of 1,919 individuals) who was cancer-free at the age of 48 years. *NBEAL1* encodes a protein that possesses two BEACH domains, suggesting a role in vesicle trafficking, membrane dynamics, and receptor signaling (Chen et al., 2004). It is expressed in multiple tissues, including breast, and is predicted to be intolerant to homozygous/double heterozygous LoF variants (ExAC pRec score = 0.96, LoF_Z score = 2.15) (Lek et al., 2016). At the somatic mutation level, *NBEAL1* point mutations and CNA are present in 2.3% of 817 BC tumors of the TCGA data set (**Supplementary Figure 2**). These mutations tend to co-occur with *BRCA2* and *TP53* mutations and are mutually exclusive with *BRCA1* mutations, although these results were not statistically significant (**Supplementary Table 12**).

### Variants Prioritized for BC Susceptibility

In addition to gene-based prioritization, 1,844 variants were prioritized through a variant-based shortlisting strategy (**Supplementary Table 10**). Variants were prioritized because they fulfilled at least one of the following criteria: a) they localized in cancer genes (CGV); b) they were shared between unrelated patients, or mapped to the same gene in unrelated patients (SVGU) or c) they were FSV. Importantly, shortlisted variants met criteria pertaining to MAF, predicted pathogenicity and presence in affected relatives (**Supplementary Figure 1**). To further shortlist candidates, we explored their presence in FBRCAX, TCGA, and UKB and report that: a) 284 were detected in FBRCAX patients, b) 97 were enriched (FET, *p* < 0.05) in NFE-TCGA patients versus NFE-ExAC-nonTCGA cancer-free individuals, c) 20 were enriched (FET, *p* < 0.05) in EUR-UKB cancer patients versus EUR-UKB cancer-free individuals, and d) 19 were enriched (FET *p* < 0.05) in EUR-UKB female BC patients versus EUR-UKB female cancer-free individuals (**Supplementary Note 9** and **Supplementary Table 13**).

Two GRBC-prioritized missense variants, *SETBP1*:c.4129G > C and *C7orf34*:c.248C > T, were present in FBRCAX and were also enriched in cancer patients from both NFE-TCGA and EUR-UKB (**Table 2**). *SETBP1* encodes a protein that binds to *SET*, a nuclear oncogene involved in DNA replication (Piazza et al., 2013). Although S*ETBP1*:c.4129G > C missense variant is predicted to have a damaging effect by only one of seven prediction tools (LRT score = 0.0001619), it was shortlisted through two strategies (CGV and SVGU), was present in two GRBC-affected sisters (diagnosed with BC at 41 and 44 years of age) and in a single FBRCAX patient. Additionally, this variant was enriched in NFE-TCGA cancer patients (OR = 1.25, 95% CI = 1.05–1.49) and in EUR-UKB female BC patients (OR = 1.13, 95% CI = 1.01–1.26). *C7orf34*:c.248C > T maps to a protein-coding gene of unknown function and is predicted to have a damaging effect by four of seven prediction tools (SIFT; Polyphen; MutationTaster; CADD). This variant was present in a GRBC mother–daughter pair (diagnosed at 60 and 47 years of age, respectively) in an unrelated GRBC patient (diagnosed at 37 years of age) and in a single FBRCAX patient. The variant was enriched in cancer patients from both NFE-TCGA (OR = 1.46, 95% CI = 1.02–2.07) and EUR-UKB cancer patients (OR = 1.14, 95% CI = 1.02–1.27).

**Table 2:** Genetic variants in GRBC and FBRCAX enriched in cancer patients from TCGA and UKB.

Gene	Nucleotide change	Protein change	rsid (v147)	GRBC carriers	GRBC families	FBRCAX carriers	Allele frequency comparisons in cancer patients vs. cancer-free individuals	Cancer patients alt allele/total alleles	Cancer-free individuals alt allele/total alleles	FET P-value	OR (95% CI)
*SETBP1*	c.4129G > C	p.V1377L	rs77518617	2	1	1	NFE-TCGA vs. NFE-ExAC-nonTCGA	169/12,388	593/54,328	0.0114	1.25 (1.05-1.49)
							EUR-UKB cancer patient vs. EUR-UKB cancer-free individuals	1,792/107,410	8,652/513,585	0.7247	0.99
							EUR-UKB female BC patient vs. EUR-UKB female cancer-free individuals	374/19,799	4,517/270,628	0.0239	1.13
*C7orf34*	c.248C > T	p.S83F	rs143475597	3	2	1	NFE-TCGA vs. NFE-ExAC-nonTCGA	44/12,392	132/54,344	0.0325	1.46 (1.02-2.07)
							EUR-UKB cancer patient vs. EUR-UKB cancer-free individuals	389/108,789	1,633/520,670	0.0216	1.14(1.02-1.27)
							EUR-UKB female BC patient vs. EUR-UKB female cancer-free individuals	63/20,110	850/274,273	0.8955	1.01(0.77-1.31)

BC, breast cancer; NFE, non-Finnish European; TCGA, The Cancer Genome Atlas; UKB, UK Biobank.

## Discussion

Associating new genes to BC susceptibility has been challenging. Although numerous sequencing-based studies, focusing primarily on candidate genes, have aimed to identify new risk candidates, over 50% HBOC cases remain of unexplained etiology, even after polygenic risk is accounted for. This is largely due to the extreme rarity of pathogenic variants, prompting some groups to suggest family-specific risk variant models for undiagnosed HBOC cases (Lynch et al., 2013). In the present study, following initial screening of patients to exclude individuals with known BC risk variants, we interrogated the whole exome of 52 Greek individuals, from 17 HBOC families for risk-associated variants. Although this is a modest-sized study, it is an important first contribution to the field, given that the Greek population is relatively understudied. In fact, a consequence of this, which is also a limitation of the present study, is the lack of allele frequency information for the Greek population, which would enable direct comparison of frequencies between Greek BC patients and cancer-free individuals. A catalogue of genetic variation is yet to be generated for the Greek population. However, given the genetic proximity of GRBC to European populations (**Figure 2**), our rationale was that risk alleles segregating at extremely low frequencies in other populations may exist in slightly higher frequencies in Greek individuals due to population-specific effects. We, therefore, sought to validate GRBC-shortlisted variants in two European ancestry French-Canadian HBOC patient groups (FBRCAX and CHUM-BC), and in TCGA and UKB cancer patients. Data sets used were from distinct studies, and thus differences in assays and data processing exist (**Supplementary Notes 2, 5, and 6**). However, given our conservative approach, which included strict filtering criteria, we do not expect the recorded differences to affect the robustness of reported findings. In fact, it is more likely that true positive findings may have been excluded as a result of the stringent approach that was applied. Given that our study focused on coding regions only, was of relatively small size, and results could not be evaluated against local allele frequencies, polygenic risk scores were not assigned in order of avoid biased or inaccurate results.

Gene- and variant-based prioritization strategies were applied to shortlist and validate GRBC-detected candidate variants. Using the former strategy, we identified LoF variants that put forth *MDM1* and *NBEAL1* as putative HBOC risk genes. *MDM1* encodes a nuclear protein that binds to and stabilizes microtubules. This protein localizes to centrioles and negatively regulates their duplication in dividing and in differentiating multi-ciliated cells (Van De Mark et al., 2015). Pathogenic variants in genes involved in the formation and function of centrosomes, such as *MDM1*, have been linked to ciliopathies, a group of diseases affecting the cilia (Hildebrandt et al., 2011). Ciliopathies have an established connection with inherited cancer syndromes, including Von Hippel–Lindau disease (VHL) (Hildebrandt et al., 2011). *NBEAL1* encodes one of nine human BEACH domain-containing proteins (BDCPs) (Cullinane et al., 2013). BDCPs play a role in molecular mechanisms including vesicular transport, apoptosis and receptor signaling (Cullinane et al., 2013). Variants in these genes have been associated with diseases such as BC, prostate cancer, multiple myeloma, Chediak–Higashi syndrome, and autism (Cullinane et al., 2013). *NBEAL1* is upregulated in gliomas (Chen et al., 2004) and comprises the least studied of human BDCPs.

The latter strategy yielded two missense SNVs, *SETBP1*:c.4129G > C and *C7orf34*:c.248C > T, detected in both GRBC and FBRCAX, that were also enriched in cancer patients of European ancestry in TCGA and UKB. Considering their relatively high frequencies (gnomAD, 0.8% and 0.2%, respectively), the reported candidates are likely to be associated with a very small increment to disease risk, if any. Whereas the function of *C7orf34* has not been described to date, *SETBP1* encodes a protein that contains numerous motifs, three nuclear localization signals, and binds to the *SET* oncogene involved in DNA replication (Piazza et al., 2013). Germline pathogenic variants in *SETBP1* cause Schinzel–Giedion syndrome, a severely debilitating condition that predisposes to neuroepithelial tumors (Lehman et al., 2008). Additionally, somatic mutations in this gene have been associated with atypical chronic myeloid leukemia (Piazza et al., 2013).

We also report two additional noteworthy findings. A private stop-gain variant was detected in the third of eleven exons of *BARD1* (c.273G > A) in a cancer-free mother (> 60 years old) of a HBOC patient (diagnosed at 37 years of age). Although there is accumulating evidence linking variants in *BARD1* to BC risk (Castera et al., 2018), LoF variants in this gene have also been reported in cancer-free individuals (https://whi.color.com). We, therefore, cannot yet ascertain whether the above variant contributes to disease risk. We also report an instance where a pathogenic variant in the established cancer gene *MEN1* (c.778G > A) is present in an affected family member, but absent from an affected second-degree relative, implying possible differential genetic causality for disease within a single pedigree. These findings highlight the high levels of genetic heterogeneity in HBOC syndrome and emphasize the need for careful interpretation of pedigrees and of the impact of genetic variants.

Studies focusing on whole exomes, and more recently on whole genomes, rather than candidate genes only, are currently fueling the discovery of novel associations between genes and disease. Whole-genome sequencing studies, which also interrogate non-coding regions of the genome, are expected to uncover new variants that influence disease risk for HBOC syndrome and multiple other diseases. In addition, recent technological advances, which address underlying structural variation in the genome, are starting to reveal the presence of genetic variants that have remained largely undetected using short read sequencing technologies and that are likely to contribute to disease. However, until these advances are applied at a large scale, the findings presented here can be used to inform lists of candidate variants and genes to be screened in additional cancer patients worldwide and to be taken forward to functional studies.

## Data Availability Statement

The datasets generated for this study can be found in the European Nucleotide Archive accession number PRJEB31704.

## Ethics Statement

Informed consent was obtained from all individuals prior to genetic testing and the study was approved by the bioethics committee of NCSR Demokritos (240/EH/11.3, updated Feb. 14, 2014) in agreement with the 1975 Helsinki statement.

## Author Contributions

ASD and JR conceived the study and were in charge of overall direction and planning. SG and KR carried out lab work. ACD and SG performed bioinformatic analyses. SG, ACD, EE, and GN performed statistical analyses. SN and A-MM-M ascertained cases, and provided patient samples. BR, WF, and PT contributed to interpretation of results from FBRCAX. SG and ASD wrote the manuscript. All authors provided critical feedback and helped shape the research, analysis, and manuscript.

## Funding

This study was supported by funds from NSRF 2007-2013 grant SYNERGASIA II (SYN11_10_19) and the Stavros Niarchos Foundation.

## Conflict of Interest

The authors declare that the research was conducted in the absence of any commercial or financial relationships that could be construed as a potential conflict of interest.

## References

[B1] Awadalla P. Boileau C. Payette Y. Idaghdour Y. Goulet J. P. Knoppers B. (2013). Cohort profile of the CARTaGENE study: quebec’s population-based biobank for public health and personalized genomics. Int. J. Epidemiol. 42, 1285–1299. 10.1093/ije/dys160 23071140

[B2] Boland J. F. Chung C. C. Roberson D. Mitchell J. Zhang X. Im K. M. (2013). The new sequencer on the block: comparison of life technology’s proton sequencer to an Illumina HiSeq for whole-exome sequencing. Hum. Genet. 132, 1153–1163. 10.1007/s00439-013-1321-4 23757002 PMC4564298

[B3] Castera L. Harter V. Muller E. Krieger S. Goardon N. Ricou A. (2018). Landscape of pathogenic variations in a panel of 34 genes and cancer risk estimation from 5131 HBOC families. Genet. Med. 10.1038/s41436-018-0005-9 29988077

[B4] Chen J. Lu Y. Xu J. Huang Y. Cheng H. Hu G. (2004). Identification and characterization of NBEAL1, a novel human neurobeachin-like 1 protein gene from fetal brain, which is up regulated in glioma. Brain Res. Mol. Brain Res. 125, 147–155. 10.1016/j.molbrainres.2004.02.022 15193433

[B5] Cullinane A. R. Schaffer A. A. Huizing M. (2013). The BEACH is hot: a LYST of emerging roles for BEACH-domain containing proteins in human disease. Traffic 14, 749–766. 10.1111/tra.12069 23521701 PMC3761935

[B6] Cybulski C. Carrot-Zhang J. Kluzniak W. Rivera B. Kashyap A. Wokolorczyk D.. (2015). Germline RECQL mutations are associated with breast cancer susceptibility. Nat. Genet. 47, 643–646. 10.1038/ng.3284 25915596

[B7] Danecek P. Auton A. Abecasis G. Albers C. A. Banks E. Depristo M. A. (2011). The variant call format and VCFtools. Bioinformatics 27, 2156–2158. 10.1093/bioinformatics/btr330 21653522 PMC3137218

[B8] De La Vega F. M. Bustamante C. D. (2018). Polygenic risk scores: a biased prediction? Genome Med. 10, 100. 10.1186/s13073-018-0610-x 30591078 PMC6309089

[B9] Depristo M. A. Banks E. Poplin R. Garimella K. V. Maguire J. R. Hartl C. (2011). A framework for variation discovery and genotyping using next-generation DNA sequencing data. Nat. Genet. 43, 491–498. 10.1038/ng.806 21478889 PMC3083463

[B10] Dreijerink K. M. Goudet P. Burgess J. R. Valk G. D. International Breast Cancer In M.E.N.S.G. (2014). Breast-cancer predisposition in multiple endocrine neoplasia type 1. N. Engl. J. Med. 371, 583–584. 10.1056/NEJMc1406028 PMC4243053 25099597

[B11] Easton D. F. Pharoah P. D. Antoniou A. C. Tischkowitz M. Tavtigian S. V. Nathanson K. L. (2015). Gene-panel sequencing and the prediction of breast-cancer risk. N. Engl. J. Med. 372, 2243–2257. 10.1056/NEJMsr1501341 26014596 PMC4610139

[B12] Ferlay J. Soerjomataram I. Dikshit R. Eser S. Mathers C. Rebelo M. (2015). Cancer incidence and mortality worldwide: sources, methods and major patterns in GLOBOCAN 2012. Int. J. Cancer 136, E359–E386. 10.1002/ijc.29210 25220842

[B13] Ferlay J. Steliarova-Foucher E. Lortet-Tieulent J. Rosso S. Coebergh J. W. Comber H. (2013). Cancer incidence and mortality patterns in Europe: estimates for 40 countries in 2012. Eur. J. Cancer 49, 1374–1403. 10.1016/j.ejca.2012.12.027 23485231

[B14] Fromer M. Moran J. L. Chambert K. Banks E. Bergen S. E. Ruderfer D. (2012). Discovery and statistical genotyping of copy-number variation from whole-exome sequencing depth. Am. J. Hum. Genet. 91, 597–607. 10.1016/j.ajhg.2012.08.005 23040492 PMC3484655

[B15] Garcia-Closas M. Gunsoy N. B. Chatterjee N. (2014). Combined associations of genetic and environmental risk factors: implications for prevention of breast cancer. J. Natl. Cancer Inst. 106. 10.1093/jnci/dju305 PMC4271030 25392194

[B16] Genomes Project C. Auton A. Brooks L. D. Durbin R. M. Garrison E. P. Kang H. M. (2015). A global reference for human genetic variation. Nature 526, 68–74. 10.1038/nature15393 26432245 PMC4750478

[B17] Hildebrandt F. Benzing T. Katsanis N. (2011). Ciliopathies. N. Engl. J. Med. 364, 1533–1543. 10.1056/NEJMra1010172 21506742 PMC3640822

[B18] Kast K. Rhiem K. Wappenschmidt B. Hahnen E. Hauke J. Bluemcke B. (2016). Prevalence of BRCA1/2 germline mutations in 21 401 families with breast and ovarian cancer. J. Med. Genet. 53, 465–471. 10.1136/jmedgenet-2015-103672 26928436

[B19] Kiiski J. I. Pelttari L. M. Khan S. Freysteinsdottir E. S. Reynisdottir I. Hart S. N. (2014). Exome sequencing identifies FANCM as a susceptibility gene for triple-negative breast cancer. Proc. Natl. Acad. Sci. U. S. A. 111, 15172–15177. 10.1073/pnas.1407909111 25288723 PMC4210278

[B20] King-Spohn K. Pilarski R. (2014). Beyond BRCA1 and BRCA2. Curr. Probl. Cancer 38, 235–248. 10.1016/j.currproblcancer.2014.10.004 25497410

[B21] Konstantopoulou I. Tsitlaidou M. Fostira F. Pertesi M. Stavropoulou A. V. Triantafyllidou O.. (2014). High prevalence of BRCA1 founder mutations in Greek breast/ovarian families. Clin. Genet. 85, 36–42. 10.1111/cge.12274 24010542

[B22] Kuchenbaecker K. B. Hopper J. L. Barnes D. R. Phillips K. A. Mooij T. M. Roos-Blom M. J. (2017). Risks of breast, ovarian, and contralateral breast cancer for BRCA1 and BRCA2 mutation carriers. JAMA 317, 2402–2416. 10.1001/jama.2017.7112 28632866

[B23] Lehman A. M. Mcfadden D. Pugash D. Sangha K. Gibson W. T. Patel M. S. (2008). Schinzel-Giedion syndrome: report of splenopancreatic fusion and proposed diagnostic criteria. Am. J. Med. Genet. A 146A, 1299–1306. 10.1002/ajmg.a.32277 18398855

[B24] Lek M. Karczewski K. J. Minikel E. V. Samocha K. E. Banks E. Fennell T. (2016). Analysis of protein-coding genetic variation in 60,706 humans. Nature 536, 285–291. 10.1038/nature19057 27535533 PMC5018207

[B25] Levy-Lahad E. Friedman E. (2007). Cancer risks among BRCA1 and BRCA2 mutation carriers. Br. J. Cancer 96, 11–15. 10.1038/sj.bjc.6603535 17213823 PMC2360226

[B26] Lynch H. Wen H. Kim Y. C. Snyder C. Kinarsky Y. Chen P. X. (2013). Can unknown predisposition in familial breast cancer be family-specific? Breast J. 19, 520–528. 10.1111/tbj.12145 23800003

[B27] Lynch H. T. Silva E. Snyder C. Lynch J. F. (2008). Hereditary breast cancer: part I. Diagnosing hereditary breast cancer syndromes. Breast J. 14, 3–13. 10.1111/j.1524-4741.2007.00515.x 18086272

[B28] Mavaddat N. Michailidou K. Dennis J. Lush M. Fachal L. Lee A. (2019). Polygenic risk scores for prediction of breast cancer and breast cancer subtypes. Am. J. Hum. Genet. 104, 21–34. 10.1016/j.ajhg.2018.11.002 30554720 PMC6323553

[B29] Mcclellan J. King M. C. (2010). Genetic heterogeneity in human disease. Cell 141, 210–217. 10.1016/j.cell.2010.03.032 20403315

[B30] Michailidou K. Lindstrom S. Dennis J. Beesley J. Hui S. Kar S. (2017). Association analysis identifies 65 new breast cancer risk loci. Nature 551, 92–94. 10.1038/nature24284 29059683 PMC5798588

[B31] Nielsen F. C. Van Overeem Hansen T. Sorensen C. S. (2016). Hereditary breast and ovarian cancer: new genes in confined pathways. Nat. Rev. Cancer 16, 599–612. 10.1038/nrc.2016.72 27515922

[B32] Piazza R. Valletta S. Winkelmann N. Redaelli S. Spinelli R. Pirola A. (2013). Recurrent SETBP1 mutations in atypical chronic myeloid leukemia. Nat. Genet. 45, 18–24. 10.1038/ng.2495 23222956 PMC3588142

[B33] Price A. L. Patterson N. J. Plenge R. M. Weinblatt M. E. Shadick (2006). Principal components analysis corrects for stratification in genome-wide association studies. Nat. Genet. 38, 904–909. 10.1038/ng1847 16862161

[B34] Rahman N. (2014). Realizing the promise of cancer predisposition genes. Nature 505, 302–308. 10.1038/nature12981 24429628 PMC4975511

[B35] Richards S. Aziz N. Bale S. Bick D. Das S. Gastier-Foster J. (2015). Standards and guidelines for the interpretation of sequence variants: a joint consensus recommendation of the american college of medical genetics and genomics and the association for molecular pathology. Genet. Med. 17, 405–424. 10.1038/gim.2015.30 25741868 PMC4544753

[B36] Rivera B. Di Iorio M. Frankum J. Nadaf J. Fahiminiya S. Arcand S. L. (2017). Functionally null RAD51D missense mutation associates strongly with ovarian carcinoma. Cancer Res. 77, 4517–4529. 10.1158/0008-5472.CAN-17-0190 28646019

[B37] Robinson J. T. Thorvaldsdottir H. Wenger A. M. Zehir A. Mesirov J. P. (2017). Variant review with the integrative genomics viewer. Cancer Res. 77, e31–e34. 10.1158/0008-5472.CAN-17-0337 29092934 PMC5678989

[B38] Shakya R. Szabolcs M. Mccarthy E. Ospina E. Basso K. Nandula S. (2008). The basal-like mammary carcinomas induced by Brca1 or Bard1 inactivation implicate the BRCA1/BARD1 heterodimer in tumor suppression. Proc. Natl. Acad. Sci. U. S. A. 105, 7040–7045. 10.1073/pnas.0711032105 18443292 PMC2365565

[B39] Solomon S. Das S. Brand R. Whitcomb D. C. (2012). Inherited pancreatic cancer syndromes. Cancer J. 18, 485–491. 10.1097/PPO.0b013e318278c4a6 23187834 PMC3565835

[B40] Stamatoyannopoulos G. Bose A. Teodosiadis A. Tsetsos F. Plantinga A. Psatha N. (2017). Genetics of the peloponnesean populations and the theory of extinction of the medieval peloponnesean Greeks. Eur. J. Hum. Genet. 25, 637–645. 10.1038/ejhg.2017.18 28272534 PMC5437898

[B41] Sudlow C. Gallacher J. Allen N. Beral V. Burton P. Danesh J. (2015). UK biobank: an open access resource for identifying the causes of a wide range of complex diseases of middle and old age. PLoS Med. 12, e1001779. 10.1371/journal.pmed.1001779 25826379 PMC4380465

[B42] Teh B. T. Esapa C. T. Houlston R. Grandell U. Farnebo F. Nordenskjold M. (1998). A family with isolated hyperparathyroidism segregating a missense MEN1 mutation and showing loss of the wild-type alleles in the parathyroid tumors. Am. J. Hum. Genet. 63, 1544–1549. 10.1086/302097 9792884 PMC1377566

[B43] Terui-Kohbata H. Yoshida M. (2017). Current condition of genetic medicine for hereditary breast cancer. Mol. Clin. Oncol. 7, 98–102. 10.3892/mco.2017.1260 28685084 PMC5492822

[B44] Thompson E. R. Doyle M. A. Ryland G. L. Rowley S. M. Choong D. Y. Tothill R. W.-. (2012). Exome sequencing identifies rare deleterious mutations in DNA repair genes FANCC and BLM as potential breast cancer susceptibility alleles. PLoS Genet. 8, e1002894. 10.1371/journal.pgen.1002894 23028338 PMC3459953

[B45] Van De Mark D. Kong D. Loncarek J. Stearns T. (2015). MDM1 is a microtubule-binding protein that negatively regulates centriole duplication. Mol. Biol. Cell 26, 3788–3802. 10.1091/mbc.E15-04-0235 26337392 PMC4626064

[B46] Wu M. C. Lee S. Cai T. Li Y. Boehnke M. Lin X. (2011). Rare-variant association testing for sequencing data with the sequence kernel association test. Am. J. Hum. Genet. 89, 82–93. 10.1016/j.ajhg.2011.05.029 21737059 PMC3135811

