# Supplementary figures and images for: Exome Sequencing in BRCA1- and BRCA2-Negative Greek Families Identifies MDM1 and NBEAL1 as Candidate Risk Genes for Hereditary Breast Cancer
Source: Front Genet. 2019 Oct 18;10:1005. doi: 10.3389/fgene.2019.01005 (PMC6813924; doi:10.3389/fgene.2019.01005)

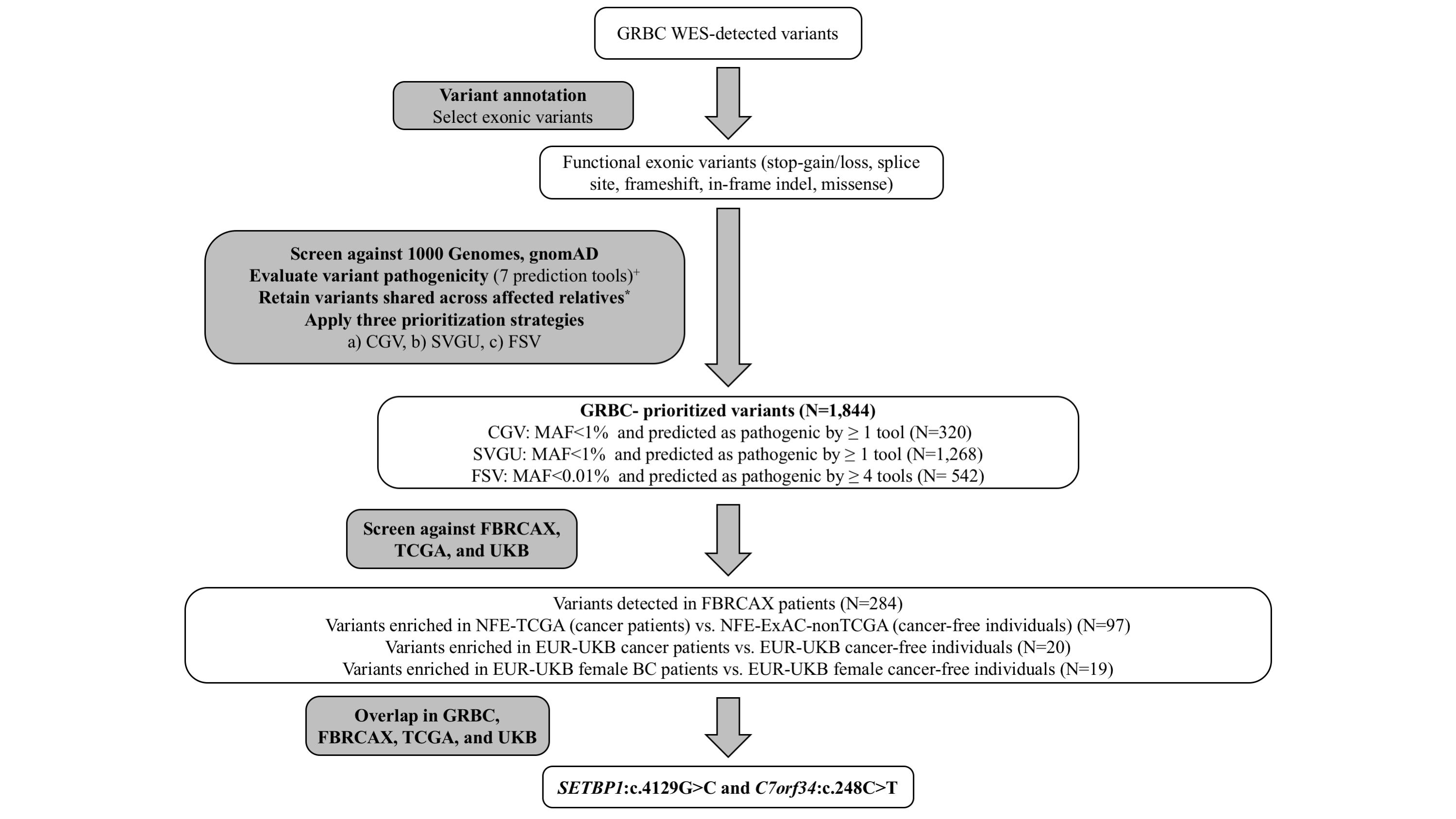

Supplement: Supplementary file 2 [file Image_1.tiff]

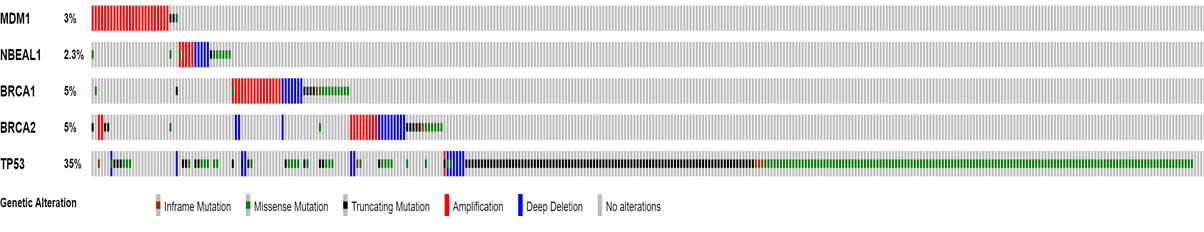

Supplement: Supplementary file 3 [file Image_2.tif]
