# Supplementary material for: Exome Sequencing in BRCA1- and BRCA2-Negative Greek Families Identifies MDM1 and NBEAL1 as Candidate Risk Genes for Hereditary Breast Cancer
Source: Front Genet. 2019 Oct 18;10:1005. doi: 10.3389/fgene.2019.01005 (PMC6813924; doi:10.3389/fgene.2019.01005)
Supplement: Supplementary file 4 [file Presentation_1.pdf]

Exome Sequencing in *BRCA1/2* Negative Greek Families Identifies *MDM1* and *NBEAL1* as Candidate Risk Genes for Hereditary Breast Cancer

Running title: Greek hereditary breast cancer candidates

Stavros Glentis[1+], Alexandros C. Dimopoulos[1], Konstantinos Rouskas[1], George Ntritsos[2], Evangelos Evangelou[2,3], Steven Narod[4,5], Anne-Marie Mes-Masson[6], William D. Foulkes[7,8,9], Barbara Rivera[7,8], Patricia N. Tonin[10], Jiannis Ragoussis[7,11]*, Antigone S. Dimas[1]*.

[1]Division of Molecular Biology and Genetics, Biomedical Sciences Research Center Al. Fleming, Vari, Greece
[2]Department of Hygiene and Epidemiology, University of Ioannina Medical School, Ioannina, Greece
[3]Department of Epidemiology and Biostatistics, Imperial College London, London, UK
[4]Dalla Lana School of Public Health, Toronto, ON, Canada
[5]Women's College Hospital, Toronto, ON, Canada
[6]Centre de recherche du Centre hospitalier de l'Université de Montréal and Institut du cancer de Montréal, Montreal, QC, Canada
[7]Department of Oncology, McGill University, Montreal, QC, Canada
[8]Lady Davis Institute, Montreal, QC, Canada
[9]Department of Medical Genetics, The Research Institute of the McGill University Health Centre, Montreal, QC, Canada
[10]Departments of Medicine & Human Genetics, McGill University; Cancer Research Program, The Research Institute of the McGill University Health Centre, Montreal, QC, Canada
[11]McGill University and Genome Quebec Innovation Centre, Montreal, QC, Canada
* Corresponding authors

Corresponding authors:
Dr Antigone S. Dimas
dimas@fleming.gr

Dr Jiannis Ragoussis
ioannis.ragoussis@mcgill.ca

+Division of Pediatric Hematology/Oncology, First Department of Pediatrics, University of Athens, Aghia Sophia Children's Hospital, Athens Greece

Supplementary Note 1

Illumina TruSight cancer panel sequencing

For initial screening of patients, the Illumina TruSight Cancer Panel was used to capture DNA regions of interest, following the manufacturer's instructions (Illumina, San Diego, USA). Indexed libraries were sequenced on an Illumina MiSeq platform using the Standard V2 kit (150 bp paired-end reads), while FASTQ, BAM and VCF files were generated through the Illumina MiSeq Reporter (Illumina, San Diego, USA). Genes and genomic regions targeted by the above panel can be found at: http://www.illumina.com/documents/products/gene_lists/gene_list_trusight_cancer.xlsx

Supplementary Note 2

DNA exome capture and sequencing

gDNA from patients who were negative for known breast cancer (BC) risk variants, or from informative relatives, was extracted from peripheral blood mononuclear cells (PBMCs) following standard salt extraction (Miller et al., 1988). Exome capture was performed on 1μg of gDNA per sample using the Ion Targetseq™ exome enrichment kit (50Mb target region) following the manufacturer's instructions (Thermo Fisher Scientific Inc., West Palm Beach, FL, USA). Enriched DNA from each individual was loaded on the Ion PI™ chip (v3) and sequenced on an Ion Proton (IP) platform (Thermo Fisher Scientific Inc., West Palm Beach, FL, USA). Seven samples (F12S01; F12S02; F12S03; F11S01; F11S02; F11S03; F11S04) were also sequenced on an Illumina platform for the purpose of comparison. For these samples, exome capture was performed on 3μg of gDNA using the SureSelect XT2 Human All Exon V5 kit (50Mb target region, Agilent Technologies, Inc., Santa Clara, CA, USA) followed by 100-bp paired-end sequencing on a HiSeq 2000 platform (Illumina, Inc., San Diego, CA, USA). Standard protocols by Agilent and Illumina were applied.

Variant calling and QC

Germline variants (single nucleotide variants (SNVs) and indels) were called from IP raw sequence data using two software packages: the IP platform built-in Torrent Variant Caller (TVC v5.0) and the Genome Analyzer Toolkit (DePristo et al., 2011) (GATK). TVC-detected variants were called using the IP plug-in for TVC with default parameters. For GATK-called variants, prior to variant calling, raw data were trimmed using trimmomatic (v0.36) (Bolger et al., 2014). Trimmed reads were aligned against Ensembl GRCh37 using BWA-mem (v0.7.7-r441) (Li and Durbin, 2010) with default options. Duplicate reads were marked using the Picard toolkit (v1.109(1716) https://broadinstitute.github.io/picard/). GATK (v3.3-0-g37228af) best practices recommendations (Van der Auwera et al., 2013) were followed to call (HaplotypeCaller) and filter (VQSR, tranche 99.5) variants. GATK was also used to call variants for the seven samples that were also sequenced on an Illumina platform. Variants with a depth of less than six reads or with GQ<20 were excluded from further analysis. We also excluded all GATK-called indels from the IP data, as these were found to have high false positive rates, in line with similar work (Zhang et al., 2015). TVC-called indels from IP data were retained. SNVs and indels passing QC filtering criteria were annotated functionally using Annovar (Wang et al., 2010). Given that CNV calling from exome data is noisy (Samarakoon et al., 2014), we retained for further analysis CNVs that were present in at least two relatives and were absent in all examined unrelated individuals. Due to differences in raw data manipulation, mapping and variant calling between the

two strategies employed (TVC and GATK), we sought to quantify the overlap between SNVs determined by each method. Additionally, for the seven samples sequenced on both the IP and Illumina platforms, we compared the overlap of SNVs and indels detected through the two different sequencing technologies.

Supplementary Note 3

Gene-based prioritization of HBOC candidate loci

For the gene-based shortlisting approach we focused on genes that harbored LoF variants (stop-gain, essential splice site, and frameshift), given their higher functional prior (McClellan and King, 2010). We identified genes that contained the same LoF variant in at least two GRBC patients from a single family, or a LoF variant in a single patient when no additional affected family members were available. We limited our search to variants with minor allele frequency MAF≤ 0.1% in both ALL 1000 Genomes and gnomAD. We then explored the presence, in FBRCAX patients, of rare LoF variants mapping in the genes prioritized as described above. We retained genes that harbored identical or different rare LoF variants in at least one FBRCAX patient and restricted our analysis to variants mapping on the same transcript. Genes were excluded from further analysis if LoF variants mapped to the last exon, as these are less likely to have a detrimental impact on gene function (Richards et al., 2015). We shortlisted as possible candidates for HBOC susceptibility genes that harbored at least one rare LoF variant in both GRBC and FBRCAX. We explored the presence of the FBRCAX-detected variants in an independent group of French-Canadian BC patients (CHUM-BC) and in control individuals (CARTaGENE).

Supplementary Note 4

Experimental validation of prioritized variants

To experimentally validate GRBC variants shortlisted through gene and variant-based strategies, we re-sequenced a subset of 148 variants, using AmpliSeq (Thermo Fisher Scientific Inc., West Palm Beach, FL, USA). Briefly, the AmpliSeq web designer (www.ampliseq.com/browse.action) was employed to design a primer pool targeting 148 variants. Selected regions were amplified from 10ng of DNA using the Ion AmpliSeq library kit (Thermo Fisher Scientific Inc., West Palm Beach, FL, USA) and amplicons were sequenced on an IP platform. Seventeen of the 148 variants were shortlisted by both gene and variant-based prioritization approaches, and 131 variants were shortlisted through variant-based prioritization. The vast majority of variants chosen for validation have not been reported in public databases to date, or have MAF<0.01% (gnomAD). In FBRCAX candidate variants were genotyped through Sanger sequencing.

Supplementary Note 5

Genotyping of FBRCAX-detected variants in CHUM-BC and CARTaGENE

Genotyping on the Sequenom iPLEX MassARRAY (Sequenom, Inc., San Diego, CA, USA) was performed to validate FBRCAX-detected variants in the two groups of French-Canadian individuals. We genotyped 512 female patients (CHUM-BC) and 1,940 cancer-free individuals (CARTaGENE, 970 female and 970 male). For CARTaGENE individuals, after excluding instances where genotyping failed, we obtained genotype information for 1,924 individuals (961 female and 963 male), and 1,919 individuals (958 female, 961 male) for the *MDM1* and *NBEAL1* variants respectively.

Supplementary Note 6

Variant allele frequencies in TCGA, ExAC and UKB

TCGA contains whole exome sequence (WES) data from ~10,000 cancer patients, including 1,000 BC cases, the majority of whom are of Caucasian ancestry (Lu et al., 2015). We approximated allele frequencies of TCGA germline variants using information from ExAC (Lek et al., 2016). ExAC is a WES database of genetic variation from 60,706 unrelated individuals, of whom 33,370 are of non-Finnish European (NFE) ancestry, from various disease and population studies. This database also includes WES data from 7,601 TCGA cancer patients, with 6,197 patients being of NFE ancestry (NFE-TCGA). The ExAC-nonTCGA dataset is a subset of ExAC in which TCGA patients have been excluded. Individuals included in the ExAC-nonTCGA dataset are from studies on phenotypes other than cancer and were thus considered to be "cancer-free" individuals. We derived NFE-TCGA genotypes by subtracting NFE-ExAC-nonTCGA from NFE-ExAC variant counts, using vcftools. Only variants with the filter status "PASS" (derived from ExAC vcf info) were selected. Also, to ensure equal representation of data from NFE-TCGA and NFE-ExAC-nonTCGA, we restricted our analysis to loci that had genotype information available for over 80% of NFE individuals in ExAC. UKB contains genotype data for 337,218 unrelated individuals of European ancestry (EUR-UKB), including 57,398 cancer patients, of whom 10,982 were diagnosed with BC (Sudlow et al., 2015). We approximated allele frequencies for EUR-UKB cancer-free individuals by subtracting variant counts for 57,398 cancer patients from the total of 337,218 UKB individuals. We also approximated allele frequencies for EUR-UKB female cancer-free individuals by subtracting allele frequencies for 10,918 BC female patients from the total of 143,844 female individuals.

Supplementary Note 7

Variant-based prioritization of HBOC candidate loci

For the variant-based shortlisting approach, we expanded the range of variant types examined in the gene-based approach to include in-frame indels, missense and stop-loss variants. For the Cancer Gene Variants (CGV) and Shared Variants/Genes in Unrelated (SVGU) strategies, we shortlisted variants with MAF<1%. Furthermore, missense variants were shortlisted if their effect was predicted to be damaging by at least one of seven prediction tools (SIFT; PolyPhen-HDIV; PolyPhen2-HVAR; LRT; MutationTaster; MutationAssessor; CADD). For the Family Specific Variants (FSV) strategy, we shortlisted variants with MAF<0.01% and damaging effects (for missense variants) as predicted by at least four of seven tools. Global population allele frequencies in 1000 Genomes and gnomAD, as well as pathogenicity prediction scores from the seven in silico tools, were assigned through Annovar. For CADD, a damaging effect was recorded for Phred scores >20. For the other tools, predictions were assigned by the software (SIFT, deleterious; PolyPhen-HDIV, probably damaging; PolyPhen2-HVAR, probably damaging; LRT, deleterious; MutationTaster, disease causing; MutationAssessor, high). For all strategies, only variants that were shared between affected relatives were considered. For SVGU we also required variants to be present in at least one unrelated patient.

Supplementary Note 8

Genes involved in cancer susceptibility and pathogenesis or in DNA repair

A list of 1,580 genes with a known role in cancer susceptibility and pathogenesis or with a role in DNA repair was compiled using information from: a) the Cancer Gene Census (http://www.sanger.ac.uk/science/data/cancer-gene-census), b) a review article focused on cancer predisposing genes (CPGs) (Rahman, 2014), c) the KEGG pathway database (http://www.genome.jp/kegg/pathway.html), d) the Human Phenotype Ontology database (human-phenotype-ontology.github.io), e) the Gene Ontology database (www.geneontology.org), and f) three sequencing diagnostic panels for cancer predisposition (BROCA panel, http://tests.labmed.washington.edu/BROCA; TruSight cancer, www.illumina.com; Qiagen cancer, www.qiagen.com). Gene names are in Supplementary Table 2.

Supplementary Note 9

Enrichment of GRBC prioritized variants in NFE-TCGA and EUR-UKB cancer patients

To explore whether variant-based prioritization candidates are enriched in cancer patients from TCGA and UKB, we compared allele frequencies between 6,197 NFE-TCGA cancer patients vs. 27,173 NFE-ExAC-nonTCGA cancer-free individuals for a total of 1,844 shortlisted variants. For EUR-UKB, given the availability of genotype data from the Axiom Array (820,967 markers), we were able to query 791 out of 1,844 shortlisted variants (42.9%).

Supplementary Note 10

Overlap of variants detected on IP vs. Illumina platforms

For seven individuals (F12S01; F12S02; F12S03; F11S01; F11S02; F11S03; F11S04), sequenced on both IP and Illumina platforms, on average 85.7% of SNVs were detected by both sequencing technologies, whereas 5.5% and 8.8% of SNVs were detected only by the Illumina and the IP platform respectively. Note that for the IP platform we considered the union of TVC and GATK-called SNVs. Indel overlap, between IP (TVC-called) and Illumina (GATK-called) variants, was at 48.8%, in line with similar studies (Boland et al., 2013) (Supplementary Tables 7 and 8).

Supplementary Figure 1. Variant-based prioritization workflow.

Supplementary Figure 2. Somatic mutation and copy number ateration (CNA) spectrum of candidate genes *MDM1* and *NBEAL1*, and of known risk genes *BRCA1*, *BRCA2* and *TP53* in 816 TCGA patients. Figure adapted from cBioPortal analysis (Cerami et al., 2012; Gao et al., 2013).

References

Boland, J.F., Chung, C.C., Roberson, D., Mitchell, J., Zhang, X., Im, K.M., *et al.* (2013). The new sequencer on the block: comparison of Life Technology's Proton sequencer to an Illumina HiSeq for whole-exome sequencing. *Hum Genet* 132**,** 1153-1163.

Bolger, A.M., Lohse, M., and Usadel, B. (2014). Trimmomatic: a flexible trimmer for Illumina sequence data. *Bioinformatics* 30**,** 2114-2120.

Cerami, E., Gao, J., Dogrusoz, U., Gross, B.E., Sumer, S.O., Aksoy, B.A.*, et al.* (2012). The cBio cancer genomics portal: an open platform for exploring multidimensional cancer genomics data. *Cancer Discov* 2**,** 401-404.

Depristo, M.A., Banks, E., Poplin, R., Garimella, K.V., Maguire, J.R., Hartl, C.*, et al.* (2011). A framework for variation discovery and genotyping using next-generation DNA sequencing data. *Nat Genet* 43**,** 491-498.

Gao, J., Aksoy, B.A., Dogrusoz, U., Dresdner, G., Gross, B., Sumer, S.O.*, et al.* (2013). Integrative analysis of complex cancer genomics and clinical profiles using the cBioPortal. *Sci Signal* 6**,** pl1.

Lek, M., Karczewski, K.J., Minikel, E.V., Samocha, K.E., Banks, E., Fennell, T.*, et al.* (2016). Analysis of protein-coding genetic variation in 60,706 humans. *Nature* 536**,** 285-291.

Li, H., and Durbin, R. (2010). Fast and accurate long-read alignment with Burrows-Wheeler transform. *Bioinformatics* 26**,** 589-595.

Lu, C., Xie, M., Wendl, M.C., Wang, J., Mclellan, M.D., Leiserson, M.D.*, et al.* (2015). Patterns and functional implications of rare germline variants across 12 cancer types. *Nat Commun* 6**,** 10086.

Mcclellan, J., and King, M.C. (2010). Genetic heterogeneity in human disease. *Cell* 141**,** 210-217.

Miller, S.A., Dykes, D.D., and Polesky, H.F. (1988). A simple salting out procedure for extracting DNA from human nucleated cells. *Nucleic Acids Res* 16**,** 1215.

Rahman, N. (2014). Realizing the promise of cancer predisposition genes. *Nature* 505, 302-308.

Richards, S., Aziz, N., Bale, S., Bick, D., Das, S., Gastier-Foster, J., *et al.* (2015). Standards and guidelines for the interpretation of sequence variants: a joint consensus recommendation of the American College of Medical Genetics and Genomics and the Association for Molecular Pathology. *Genet Med* 17, 405-424.

Samarakoon, P.S., Sorte, H.S., Kristiansen, B.E., Skodje, T., Sheng, Y., Tjonnfjord, G.E., *et al.* (2014). Identification of copy number variants from exome sequence data. *BMC Genomics* 15, 661.

Sudlow, C., Gallacher, J., Allen, N., Beral, V., Burton, P., Danesh, J., *et al.* (2015). UK biobank: an open access resource for identifying the causes of a wide range of complex diseases of middle and old age. *PLoS Med* 12, e1001779.

Van Der Auwera, G.A., Carneiro, M.O., Hartl, C., Poplin, R., Del Angel, G., Levy-Moonshine, A., *et al.* (2013). From FastQ data to high confidence variant calls: the Genome Analysis Toolkit best practices pipeline. *Curr Protoc Bioinformatics* 43, 11 10 11-33.

Wang, K., Li, M., and Hakonarson, H. (2010). ANNOVAR: functional annotation of genetic variants from high-throughput sequencing data. *Nucleic Acids Res* 38, e164.

Zhang, G., Wang, J., Yang, J., Li, W., Deng, Y., Li, J., *et al.* (2015). Comparison and evaluation of two exome capture kits and sequencing platforms for variant calling. *BMC Genomics* 16, 581.
